# Combining patient, clinical and system perspectives in assessing performance in healthcare: an integrated measurement framework

**DOI:** 10.1186/s12913-019-4807-5

**Published:** 2020-01-08

**Authors:** Jean-Frederic Levesque, Kim Sutherland

**Affiliations:** 1 Agency for Clinical Innovation, 67 Albert Avenue, Chatswood, New South Wales 2067 Australia; 2 Centre for Primary Health Care and Equity, University of New South Wales, Randwick, New South Wales 2052 Australia

**Keywords:** Performance measurement, Conceptual framework, Quality improvement

## Abstract

**Background:**

The science of measuring and reporting on the performance of healthcare systems is rapidly evolving. In the past decade, across many jurisdictions, organisations tasked with monitoring progress towards reform targets have broadened their purview to take a more system-functioning approach. Their aim is to bring clarity to performance assessment, using relevant and robust concepts – and avoiding reductionist measures – to build a whole-of-system view of performance. Existing performance frameworks are not fully aligned with these developments.

**Methods:**

An eight stage process to develop a conceptual framework incorporated literature review, mapping, categorisation, integration, synthesis and validation of performance constructs that have been used by organisations and researchers in order to assess, reflect and report on healthcare performance.

**Results:**

A total of 19 performance frameworks were identified and included in the review. Existing frameworks mostly adopted either a logic model (inputs, outputs and outcomes), a functional, or a goal-achievement approach. The mapping process identified 110 performance terms and concepts. These were integrated, synthesised and resynthesised to produce a framework that features 12 derived constructs reflecting combinations of patients’ needs and expectations; healthcare resources and structures; receipt and experience of healthcare services; healthcare processes, functions and context; and healthcare outcomes. The 12 constructs gauge performance in terms of coverage, accessibility, appropriateness, effectiveness, safety, productivity, efficiency, impact, sustainability, resilience, adaptability and equity. They reflect four performance perspectives (patient, population, delivery organisation and system).

**Conclusions:**

Internationally, healthcare systems and researchers have used a variety of terms to categorise indicators of healthcare performance, however few frameworks are based on a theoretically-based conceptual underpinning. The proposed framework incorporates a manageable number of performance domains that together provide a comprehensive assessment, as well as conceptual and operational clarity and coherence that support multifaceted measurement systems for healthcare.

## Background

Across healthcare systems in high income countries, there is an established consensus that independent and impartial assessment of performance is an essential part of quality improvement efforts. Organisations and agencies that specialise in healthcare performance measurement and reporting act to oversee system functioning, promote accountability, highlight variation, identify areas for improvement, and make information available to leverage and support change [1, 2].

There is a growing recognition of the important role played by public reporting in healthcare [3, 4]. It confers positive effects as a lever for improvement but also has potential for negative unintended consequences, such as gaming or a blinkered preoccupation with a small number of published, often easily measurable, metrics. The power of public reporting means there is an imperative to accurately, fairly and meaningfully measure and report comparative information. Given the complexity of healthcare systems, this is a real challenge. Healthcare services – the principal subject of performance reporting efforts - are shaped, directly and indirectly, by a wide array of organisations and professionals working with patients. There is a huge variety and volume of tasks undertaken to diagnose, deliver, support, guide, and assure provision of care that improves peoples’ health.

Clinicians’ sensitivity to comparative data and the strong debates that media coverage can generate means that reporting must be comprehensive, systematic and rigorous. Seeking breadth and comprehensiveness in performance reporting has seen a burgeoning of measures that reflect different aspects of performance in complex systems. However, this has contributed to what the Institute of Medicine has called ‘indicator chaos’, suggesting that there are too many indicators, and poor delineation [5]. Paradoxically, at the same time, there are concerns that indicators or concepts have been too narrowly focused and these have led to calls for broader and more expansive measurement of outcomes and value [6, 7].

This paradox may in part be a reflection of the absence of a clear definition of high performance [8] and a lack of conceptual clarity about how to assess performance domains within complex adaptive systems. Frameworks feature in many settings and have been used to guide public reporting efforts [9–19]. These existing frameworks have been successful in sorting and classifying different metrics into related thematic areas such as access, patient-centeredness, safety or efficiency. They are often a reflection of whatever data are available and the particular aspects of healthcare delivery that are the focus of current policies or priorities.

However, few are clearly grounded in theory or make explicit links between conceptualisation and operationalisation of performance measurement [1, 20, 21]. Many existing frameworks are populated through the use of a Delphi process to select healthcare quality indicators [22]. While useful and insightful for many applications, the use of Delphi processes for indicator selection or for framework construction – does not necessarily result in a clear, conceptually sound result.

This paper seeks to apply a theoretically grounded approach to performance framework development - drawing on similar efforts to link multidisciplinary bodies of knowledge in non-health contexts. Jabareen [23] refers to a conceptual framework as a network of interlinked concepts that together provide a comprehensive understanding of a phenomenon or phenomena and asserts that building such a framework is an iterative process – one that requires an understanding of the relationships between the concepts that provide the building blocks for the overall framework. “A conceptual framework is not merely a collection of concepts, but rather a construct in which each concept plays an integral role” (p51). We used such an approach to inform the creation of comprehensive, conceptually grounded measurement systems.

## Methods

**Fig. 1:**
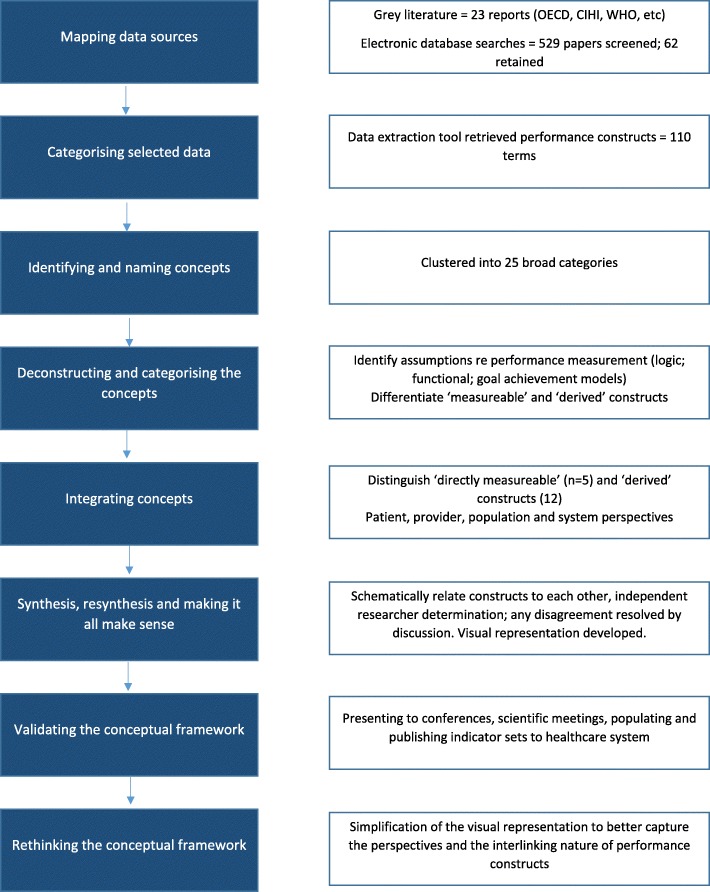
Schematic of the framework development approach

An eight-phase approach, described by Jabareen (2009), guided the framework development (Fig. 1). These eight phases are: 1) Mapping selected data sources; 2) Categorising of the selected data; 3) Identifying and naming concepts; 4) Deconstructing and categorising the concepts; 5) Integrating concepts; 6) Synthesis, resynthesis and making sense; 7) Validating the conceptual framework; 8) Rethinking the conceptual framework. While elements of our approach resonate with those of a scoping review (24, 25) our purpose was not to map the available evidence, but to collect the range and distribution of concepts which have been used to measure performance and their theoretical basis.

A review of the academic and grey literature identified concepts and frameworks related to the measurement of performance in healthcare. A targeted search of the websites of performance reporting agencies at international, national and, when appropriate, subnational levels; jurisdictional health ministries or departments; and major health services research organisations was supplemented by a rapid literature review based on searches of the Database of Abstracts of Reviews of Effectiveness (DARE); Cochrane Database of Systematic reviews; and PubMed electronic data base. Search terms were: “conceptual framework” AND performance; “concept map*” AND performance; “performance framework”. PubMed searches (to June 2017) identified 648 articles.

Citations were screened for suitability for inclusion by one author (KS). A total of 62 articles and 27 grey literature reports fulfilled the selection criteria (i.e. described a conceptual framework or model that sought to measure, assess and / or report on performance in high income healthcare systems). Papers and reports simply using or citing another framework were not retained (Appendix 1).

A bespoke data extraction tool was developed and applied by one author (KS) to capture relevant terms and constructs used in each of the retained frameworks, articles and reports. These were clustered independently by both authors into broad categories – combining related terms such as access, accessibility, access to care, affordability. Any differences in categorisation were resolved via discussion. Using an interpretive review approach [24], the broad categories were critically assessed in terms of underlying assumptions and the extent to which the constructs are directly measurable. Concepts were then categorised according to whether they reflect patient, provider, population or system perspectives. Interdependencies and relationships between the concepts were described through a process of synthesis by individual researchers independently, followed by comparison and resolution; independent resynthesis followed by comparison and resolution. A visual representation was then developed iteratively.

## Results

A total of 19 performance frameworks were identified and included in our review (see Additional file 1) [5, 9, 10, 12, 13, 17, 21, 25–38]. The content of each framework was analysed with regards to the performance constructs mentioned. A total of 110 different distinct terms were featured in the frameworks. These were clustered into 17 broad concept groups (see Appendix 2). The most commonly used concepts were ‘appropriateness’ (featured in 19 frameworks), ‘efficiency’ (15), ‘safety’ (14), ‘accessibility’ (13), ‘equity’ (12), ‘impact’ (11) and ‘effectiveness’ (11). In addition to the wide variety of terms or constructs featured in the performance frameworks, there was also variation in the extent to which they included directly measurable or derived or derived constructs. Our mapping exercise identified three approaches that have been used to underpin performance measurement efforts: logic models, theory-based models and goal achievement models.

### Typology of frameworks of performance measurement

The first set of frameworks conceptualise performance to be relating inputs, activities, outputs and outcomes. These models look at flows of production [37] and logic models [38, 39]. These models often build on the structures, processes and outcomes of healthcare categorisation proposed by Donabedian [40] where structure describes the settings where care is delivered and the physical, human and financial resources required. Process refers to patient and practitioner activities involved in giving and receiving care and outcome describes the effects of care in terms of changes in health status, patient’s knowledge and/or behaviour; and patient satisfaction.

The second set of frameworks are more theoretically based and conceptualise performance in terms of functions or roles within systems. These models draw on Parson’s theory of social action [41] where performance is seen as achievements in the functions of adaptation, goal attainment, production and values maintenance [42, 43]. In this paradigm, the alignment and balance of these key functions is the primary concern, rather than the actual relationships between inputs and outputs.. It defines adaptation as the ability to secure resources, shape structure, systems and processes according to community needs. Goal attainment is here defined as achievement of targets relating to population health and equity. The production function relates to the quantity and quality of services. Finally, the value maintenance function refers to how systems maintain their capacity and continually develop and evolve.

The third set of frameworks assess performance in terms of societal goal achievement. These models are conceptually agnostic and depend on the definition and codification of a set of values, standards or objectives against which performance is to be judged. These models are grounded in the organisational literature on scientific management, goal setting [44] and management by objectives [45], and consider socially determined goals as core to performance and the strategic orientation required to assess performance. Within this paradigm, assessment of performance involves evaluating the extent to which goals are realised or achieved.

### Introducing an integrated measurement framework of performance

Building upon the conceptual antecedents in existing performance frameworks described above, our framework integrates all three of these approaches (See Table 1 and Additional file 2). This new framework builds on a logic model base – but moves away from simplistic ‘counts’ of needs, resources, activities or outcomes - recognising that increases or decreases in any of these do not necessarily correspond to an improvement or deterioration in performance. It also incorporates functional aspects of healthcare systems with derived constructs such as accessibility and effectiveness and safety, and encapsulates goals of the healthcare system such as equity and impact. Crucially, it acknowledges complexity and dynamism and considers performance using a perspective that relates measurable elements to each other (e.g. patients’ needs and expectations with activity) allowing for more meaningful judgements to be made about performance in context and across different time horizons.

**Table 1:** Measurable, functional and goal-oriented constructs

Constructs	Key question / theme	Example of indicators
1. Measurable constructs		
Patients’ needs and expectations What is needed? In what format is it needed?	Patients’ need for healthcare can be quantified in measures of ill health, prevalence of chronic illness, limitations to daily activities caused by health issues, or health literacy. Expectations can relate to personal interactions (courtesy, engagement), facilities (e.g. buildings, equipment, staff), processes (e.g. waiting lists, accessing care), and health outcomes (e.g. the anticipated effects on patients’ health).	Patients’ needs: Number of people in poor health; Self-reported health status; Prevalence of diabetes; Health literacy. Patients’ expectations: Importance of politeness and courtesy; Perception of delays or waiting times; Desire for choice and engagement in care decisions.
Healthcare resources and structures What is invested in healthcare? How is it configured?	The investments in, allocation and organisation of healthcare resources. It includes the tangible inputs to the healthcare system and the way they are ordered and managed such as financial and human resources, equipment, buildings and organisational hierarchies. More intangible aspects include culture and the symbolic structures established, such as values and organisational norms.	Healthcare resources: Number of doctors, nurses; Financial and human resources invested. Healthcare structures; Organisational models of care; Organisational climate and culture; Allocation models.
Healthcare services What type and how many services are delivered? In what manner are they delivered?	Counts and attributes of the goods and services provided to patients. The core activity of healthcare providers, this construct includes consultations, surgeries, pharmaceuticals, diagnostic tests, and treatments (the amount of care provided). In addition, it includes the characteristics of the service provided (the way care is provided).	Healthcare services: Number of surgeries; Number of emergency department visits; Receipt of care; Healthcare quality: politeness; respect; precision; consistency.
Healthcare processes, functions and context How is healthcare organised? How is it functioning?	A focus on standard operating practices and how various components of the system interact together during the process of delivering services. This includes many sub-constructs related to the flow of services and information, and interactions between professionals and other providers, and between providers and the broader context they operate within.	Healthcare processes: Models of care; Patient pathways and protocols; Coordination and integration processes; Flow of information; Collaboration.
Healthcare outcomes Have needs been fulfilled? Have expectations been met?	A focus on health and wellbeing. Metrics are often based on patient reported measures and activities of daily living. Includes physical, psychological, social effects of care and maybe also the outcomes that are generated by experience of care such as trust and confidence in capacity to manage care.	Number of deaths per 100,000 population Number of healthcare associated infections Health-related quality of life measures
2. Functional and relational constructs of performance – Patient perspective – accessibility, quality and outcomes		
Accessibility Is healthcare provided when, by whom and where needed? Is healthcare provided at the expected cost and time?	The extent to which patients are able: to recognise and identify their healthcare needs; to seek care; to reach providers of care; to pay for care; and to receive care that is proportionate and matched to their needs. Metrics quantify whether services can be easily sought, reached, obtained and adhered to. Includes sub-constructs: affordability; availability of services; timeliness; unmet needs; organisational accommodation; social and cultural acceptability.	Out of pocket costs Number of visits relative to number of expected visits Patient survey data measuring reported barriers to care Waiting times / timeliness / punctuality
Appropriateness Is the right healthcare provided, in the right way, and in the right amount?	The extent to which patients receive services that respond to: a) their health needs, b) align with best-practice models of care; c) is delivered in a technically proficient way; d) in accordance with their expectations about the manner in which they should be treated	Compliance with recommended care (e.g. proportion of AMI patients discharged on preventive medications) Patient survey data on patient-centredness
Safety Is care provided in a way that prevents harm and does not cause harm to patient?	Incorporates the notion of risk – are processes in place to prevent unnecessary harm to patients –both minimising iatrogenic harm and acting in a way that interrupts patient deterioration and circumvents exacerbations that are amenable to care.	Hand hygiene or surgical checklists compliance Infection control Adverse events
Effectiveness Does healthcare make a positive difference to patients’ health? Are needs of patients reduced? Is disease progression altered?	The extent to which healthcare services deliver to patients the benefits expected. Measurement assesses whether services reduced the incidence, duration, intensity or consequences of patients’ presenting health problem. Metrics include risk standardised mortality and readmission rates, as well as patient confidence in providers and the broader system.	Patient reported outcome measures Relative survival Symptom control Changes in activities of daily living
3. Functional and relational constructs of performance – static system perspective		
Coverage Are healthcare resources and structures established according to needs and expectations?	The extent to which services rendered meet the potential need for those services in a community.	Schedule of available funded procedures and treatments Patient reported confidence in ability to access care Consequences of unmet need (e.g. dental caries)
Productivity Does the healthcare system produce sufficient quantity and quality of care for the resources invested?	The number of goods and services delivered per unit of resource. Often referred to as technical efficiency.	Consultations per physician Scans per CT facility Cost per bed day
Efficiency Does the system achieve good outcomes and patients’ experiences for the resources invested?	The extent to which healthcare systems and organisations make the best use of available resources. Assessed by quantifying the amount of valued outcomes achieved for the resources invested. The definition of valued outcomes is important– more services per unit of input are not necessarily desirable. Metrics focus on value for money; or conversely on waste, duplication and unnecessary care. Relates to allocative and technical efficiency.	Unnecessary duplication of tests Number of consultations per doctor Relative stay index
4. Functional and relational constructs of performance – dynamic system perspective		
Adaptability Does the system adapt to changing patients’ needs and expectations across diverse contexts of delivery?	As the demands for healthcare services - and the technologies available to deliver them - change, systems need to be able to adapt to respond, and planning tools need to recognise the interdependencies within the care service and care infrastructure system.	Shifts in supply patterns in response to health trends Uptake rates of effective new technologies Introduction of new models of care to meet emerging expectations
Sustainability Is the quantity and quality of care sustainable in future years? Can the system continue to work at this level of performance?	The extent to which healthcare systems function in ways that meet patients’ current health and healthcare needs without compromising the ability to meet needs in the future. Sustainable systems and organisations adapt to changing circumstances, constraints, opportunities and demands. There are very few direct measures of sustainability and so assessment focuses on quantifying the use of processes proven to improve efficiency, impact and productivity	Investment in Research & Development programs Utilisation of cost effective alternative models of care Pace of increase in expenditure Absenteeism, long term vacancies, use of locums Assured supply of essential drugs
Resilience Can care health outcomes be maintained in the face of unexpected changes and challenges?	At an organisational and system level, resilience is the ability to mount a robust response to unforeseen, unpredicted, and unexpected demands and to resume or continue normal operations. Metrics often focus on disruptions in the continuity of care as indicators of the inability of systems to meet demand. Gap-filling adaptations such as clinician initiatives and improvements to equipment design indicate sources of resilience that are present to help accommodate demands for care.	Flexibility – ability to mobilise resources when required Timeliness in high activity periods in the emergency department Elective surgery cancellations when there is heightened demand for emergency surgery
5. Goal attainment constructs of performance – population perspective		
Impact Protecting, promoting and promulgating health	The influence that services have on a population’s overall health and functioning. This construct includes measures of change in public health, or trends in terms of changes in quality of life and wellbeing. Impact measures reflect complexity, the integration of care and the cumulative effect of discrete events, and of health promotion, preventive or curative interventions.	Premature mortality Life expectancy Activities of daily living Changes over time in health status
Equity Fairness in health, fairness in healthcare	The extent to which everyone in a population has the opportunity to reach their full health potential, equity incorporates the idea that receipt of care, appropriateness of care and outcomes of care should be consistent across social groups and responsive to needs. Equity is not synonymous with equality – but includes the notion of ‘fairness’ – those with greater need should get more care. Horizontal equity refers to the provision of equal healthcare to those who have the same need, regardless of other personal or social characteristics. Vertical equity involves treating population sub-groups differently, according to differential need.	Disparities in accessing care for equal need Infant mortality by Aboriginality

The integrated framework proposes five measurable constructs (patients’ needs and expectations; healthcare resources and structures; receipt and experience of healthcare services; healthcare processes, functions and context; and healthcare outcomes) which are generally used to populate logic model approaches. These five elements are the aspects of healthcare performance that can be directly measured through quantitative data collection systems or approaches.

**Fig. 2:**
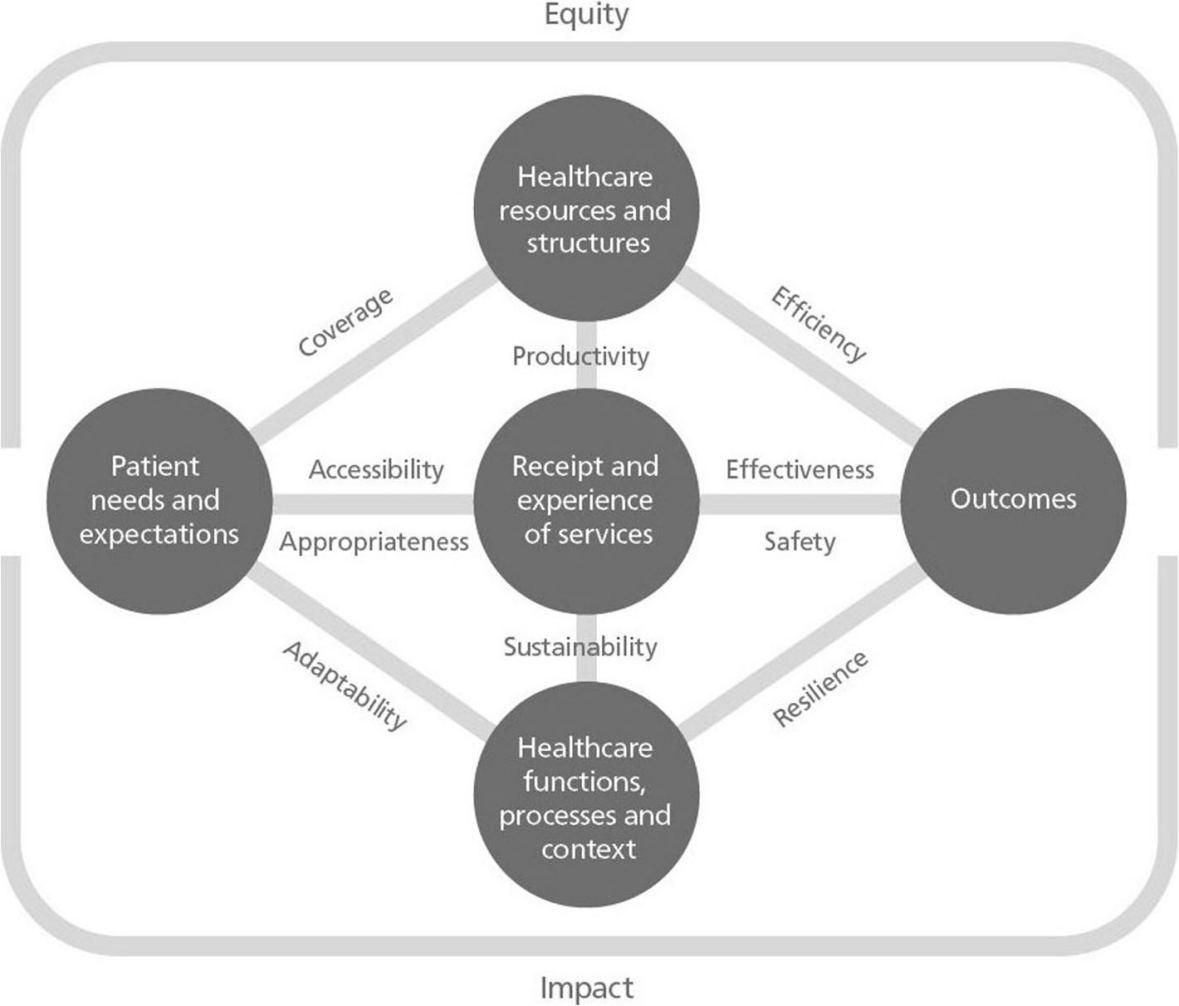
Integrated performance measurement framework

Building on these measurable constructs and encapsulating a functional approach, the framework identifies 10 derived constructs of performance (coverage; accessibility; appropriateness; safety; effectiveness; productivity; efficiency; adaptability; sustainability; resilience). The framework also incorporates two overarching derived constructs that relate to goal achievement (population health impact; equity) – recognising their importance in many healthcare systems. Equity is an overarching construct that relates to the population distribution of other constructs, such as access, appropriateness and effectiveness. Similarly, impact is an overarching construct determined by the cumulative contribution of all other constructs. While these are the 12 key constructs of performance, their measurement requires the combination of the previous five measurable constructs as they cannot be measured directly (e.g. to derive efficiency measures, quantification of resources expended is assessed in relation to quantification of outcomes achieved.) Figure 2 illustrates the relationship between measurable and derived constructs of performance.

Table 2 summarises the inclusion of constructs in selected frameworks from the literature. From this table we can see very few frameworks are theoretically based but those that are tend to be more comprehensive in construct inclusion.

**Table 2:**
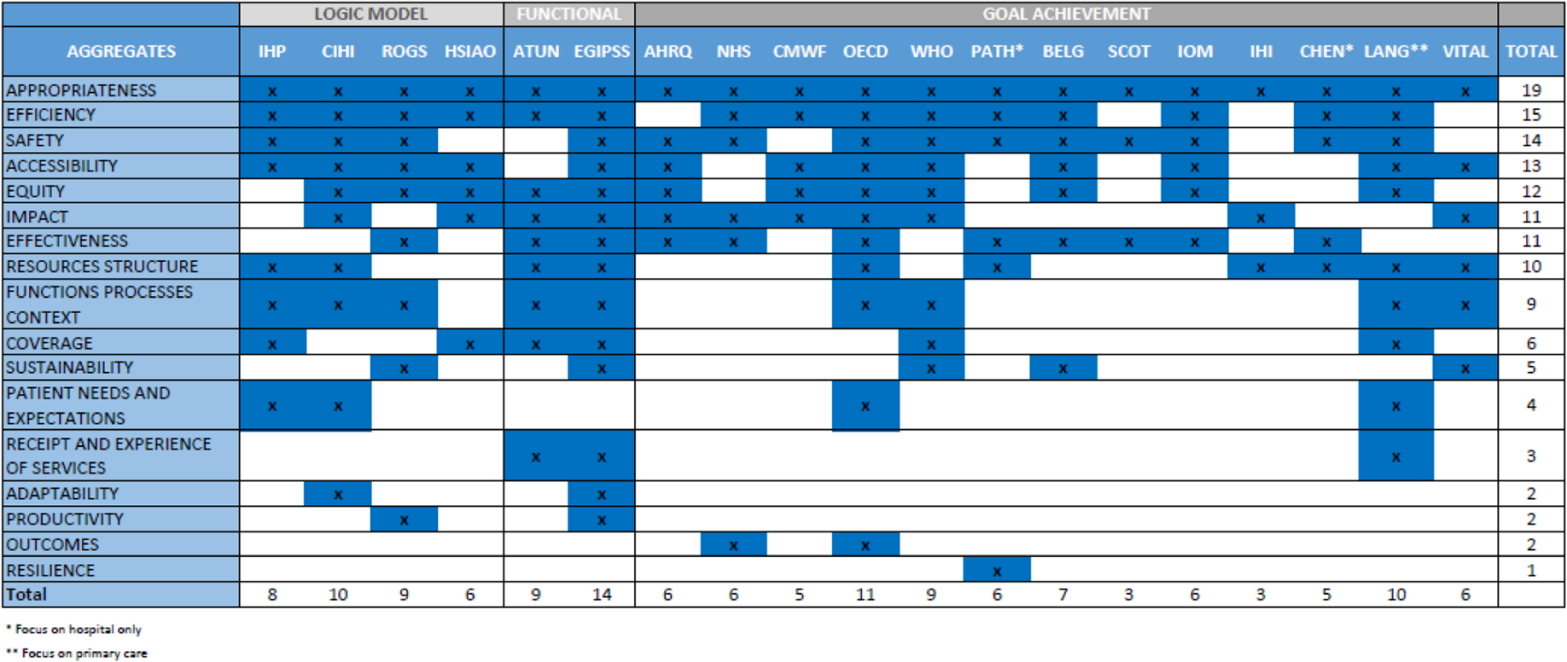
Inclusion of constructs in selected frameworks from the literature

## Discussion

Looking across existing performance assessment frameworks, it is clear that there is a core set of constructs that resonate across contexts and jurisdictions. Topics such as access, safety, quality, responsiveness, effectiveness and productivity all feature prominently in the existing literature. However we found very few frameworks that are structured in line with an underlying theory or a conceptual framework that can be applied empirically to capture the entire breadth of constructs required to understand healthcare performance.

We constructed a conceptual framework of healthcare performance measurement that relies on logic, theorisation and mapping. It revealed two key principles. The first principle is that performance is a relative construct – it reflects an outcome in relation to a need; a tangible change in relation to context; a benefit in relation to a cost. While this seems self-evident, it is not always the basis on which healthcare performance is measured and reported. Very often, measurable constructs such as number of hospitalisations, procedures or beds are used to populate indicator sets. More meaningful assessment requires derived metrics that place various elements of healthcare delivery relative to others. For example, it is not possible to directly measure accessibility – rather, patients’ receipt of services has to be measured and considered relative to their needs and expectations.

The second principle is that performance is multi-layered and therefore a contested construct. It is often referred to in terms of ‘value’ or ‘quality’ [26] – notions which can differ across patient, provider, system and population perspectives. Again, this is not a revolutionary assertion but to date, it has not been fully encapsulated in healthcare performance frameworks.

The horizontal axis of the model encapsulates patients’ perspectives – it represents the notion of right care, right time, right way, and right amount. To assess performance from a patient perspective, we develop measures that relate in various combinations: patients’ needs and expectations, services received, and outcomes. The model shows there are two key parallel constructs linking patient related measurable elements. ‘Patient needs and expectations’ and ‘receipt and experience of services’ are bridged by accessibility and appropriateness – reflecting respectively whether any care was received and whether that care was proportionate and tailored to patients’ needs and expectations.

In the central vertical axis, the model considers value from a delivery or organisational perspective – spanning resources and structures (classically referred to as inputs), services provided (outputs); and functions, processes and context (where functions refer to key deliverables such as health promotion, processes refer to priority setting and assurance; and context refers to broader elements such as social determinants of health). The derived constructs are productivity and sustainability.

The framework reveals how apparently similar indicators can in fact reflect different constructs of performance. Indicators are shaped by the measurable components they draw upon and there are multiple combinations or permutations possible. For example, while the number of patients who received a specific intervention is purely a descriptor of receipt of care, once this measure is linked with the actual number of patients requiring this intervention, it then reflects on accessibility, while if it is related to the change in patients’ health status, it is then a measure of effectiveness.

The framework also highlights that there can be reinforcing and antagonistic relationships and feedback loops between constructs that change over time. This may help explain observed unintended consequences of performance reporting [46]. Such consequences may reflect efforts that oversimplify performance assessment by describing what happened in healthcare (e.g. volume and attributes of services provided) but fail to consider these in relation to each other –thereby missing an opportunity to generate understanding (e.g. revealing accessibility by combining the volume of services provided with the number of people requiring it). The derived constructs are logically linked together. Reinforcing relationships exist – for example between efficiency and coverage – where finite resources are not wasted, there is potential for greater coverage; or between the delivery of appropriate care and resulting effectiveness and impact; or between gains in accessibility and the achievement of more equitable healthcare. Reinforcing loops can also act in concert where weak performance in one construct has a dampening effect on other constructs. For example, low efficiency equates to fewer available resources, fewer activities, less coverage; ineffective treatments clearly represent inefficient care; and poor coverage leads to reduced equity and impact.

Conversely, strong performance in one domain can have an antagonistic or detrimental effect on another. For example, over-emphasis on effectiveness can come at a heavy cost - many innovations and new therapies can entail high costs for marginal incremental benefits and therefore might reduce overall efficiency; or high levels of efficiency may only be achieved at the expense of population groups that are more difficult to reach and have less chance of benefitting from treatments, reducing the equity of the system; or maintaining very high levels of appropriateness and responsiveness in some clinical areas might reduce a system’s capacity to ensure a widespread coverage.

The complexity of the interplay between constructs of performance is further heightened when temporality is considered. For example, accessibility that influences performance in a baseline year can affect impact in future years. In a complex dynamic system such as health, maximising the results in any single construct is difficult, if not impossible, to achieve. Even if it were possible, it is not desirable. Given their interdependencies, maximising one construct would likely have unintended consequences on others. Measuring them simultaneously is therefore very important [47].

### Towards a ‘measurement system’

The notion of a measurement system has been present in the broader management literature for 50 years [48]. Much of this work clearly differentiates measurement that is for system or performance management purposes from measurement that is for benchmarking and improvement purposes [49]. In health, this is a key distinction with Ministries or Departments of Health often focused on performance management while agencies mandated to secure quality improvement and clinical innovation are more focused on identifying areas of variation in the delivery of healthcare to patients and ways to address them.

The proposed framework can support more comprehensive assessment and also provide transparency about decisions regarding which aspects of performance are measured and reported. Its principal purpose is measurement – although it has clear relevance for quality improvement and for policy.

Healthcare system performance reporting efforts to date have generally featured a preponderance of the simple measurable elements (e.g. utilisation) and a lack of measurement of derived constructs (e.g. organisational functioning). In populating frameworks with metrics, indicators and data, most systems have used a pragmatic approach, focusing on readily available measures and aspects that are relevant to specific policies and contexts, but remain mute about important aspects of performance. In some cases, this has led to sets of disparate indicators that are unable to provide a comprehensive picture of performance. There is a growing recognition that performance measurement efforts should move beyond opportunistic and piecemeal approaches to indicator selection and towards deliberative filling of information gaps [50].

Conceptual clarity does not mean that a handful of measures will do. Over-reliance on a very small number of metrics infers a strong correlation of performance across all constructs, whether measured or not. However, this has not been supported empirically and a number of studies report only weak correlations between different metrics [51–53]. In other words, performance in one construct of healthcare is not necessarily informative about performance in others [54].

Overall, we found that most performance frameworks lack a theoretical basis. The lack of theory has perhaps led to a proliferation of measurement scorecards and frameworks that are based on empiricism. This empirical base has meant what is measurable features in many performance framework efforts. More theory or conceptual clarity may mean more parsimony – we don’t need every permutation of measurement to get an understanding of the measurement balance between aspects of performance – what we need is a well-constructed conceptually sound model that can be used for a range of purposes - measurement, quality improvement and policy – and in a range of contexts.

Some frameworks currently in widespread use provide only a partial picture of performance – albeit a critically important one. For example the Institute for Health Improvement’s hugely influential “triple aim” [32] encapsulates elements of appropriateness (experience of care), efficiency (per capita cost) and impact (population health) but it provides a partial view of performance – and while its focus attracts attention with its simplicity, it can be considered to be somewhat reductive – overlooking essential elements of performance, such as accessibility or equity. Simple models can resonate but the trade-off is a loss of sensitivity to complexity and measurement. Paradoxically – more conceptually grounded frameworks may appear piecemeal – because they may have empty categories – our ability to quantify with data may not be advanced sufficiently to fill all the conceptual categories. It does however allow for future proofing and guide data collection and analysis efforts.

Similarly, there is a clear tension between trying to summarise whole health system performance in a ‘single number’ measure and juxtaposing fragmentary metrics that inform about parts of the system [1]. Trying to integrate measures of performance into a single score is likely to prove to be a meaningless task. Embracing the complexity of performance with a framework that enables a clearer assessment and understanding of which data can inform the constructs of performance to be assessed, and how they relate to each other, is more productive than trying to oversimplify performance. For example, understanding trends in the needs of population allows better understanding of why coverage may be decreasing; and reflects on adaptability. In assessing overarching constructs such as equity – sophisticated performance measurement approaches are able to reveal how disparity may be explained by another construct, such as coverage (e.g. unwarranted variation in receipt of surgery by socioeconomic status, as a result of resource allocation that does not match needs for surgery).

### Limitations of the framework

The framework provides a clear set of principles for measuring performance. While it is represented by a simple visualisation, it encapsulates considerable complexity and requires substantial effort to be populated with a comprehensive set of indicators. This breadth means that the framework may not be best suited to target efforts or focus a system on key current problems. The metrics highlighted in the paper are quantitative in nature however, ‘soft intelligence’ or experiential evidence is increasingly considered to be an important additional source of performance information - capturing perspectives of actors and sensitivities to context [55]. Because we have distilled the variety of terms used in many frameworks, policy makers and clinicians might be unsettled not to see some classic constructs such as ‘quality’ that have previously been promoted. The review adopted neither a systematic review nor a scoping review method. This was because its purpose was to collect the range and distribution of concepts that have been used to measure healthcare performance and their theoretical basis and the more conceptually grounded approach of Jabareen better aligned with that purpose. Having said that however, the search phase was extensive and seminal reports and papers have been used in a snowball approach to access other key references and ensure an exhaustive review. Finally, because of the salience of performance measurement mostly in high income countries, the framework produced is mostly applicable to these settings and its transposition to other healthcare settings remains to be assessed.

## Conclusion

Our proposed framework encapsulates well used concepts seen in many previously published frameworks. While much of it seems familiar, it does involve a fundamental shift in thinking about performance assessment and its conceptualisation - bringing explicit recognition of the complexity and interconnectedness that constitutes performance. The framework provides a means to resolve what sometimes appears to be “indicator chaos” by identifying 12 clearly defined constructs of performance that synthesise over 100 different constructs used previously and can be used to reflect different perspectives and roles.

Focusing on derived aspects of performance drives assessment efforts beyond description. The proposed framework leverages constructs used widely in other frameworks (e.g. accessibility, appropriateness, effectiveness) [18] but where previous efforts have often considered elements of performance in isolation, the framework proposed here uses measures that are dynamic, sensitive to context, and to interlinked processes in healthcare delivery. Moving forward, this approach can help highlight current gaps in indicators and drive the development of measures that truly reflect performance – moving beyond simplicity to insight – combining different pieces of data to develop a more meaningful measure of performance, and one that does not focus solely on outcomes.

Performance as a concept can be beguilingly simple. Similarly, the proposed framework is at first glance, visually simple. However both performance and the framework are multilayered and complex, shaped by actions, reactions and interactions in an interconnected network. Performance is difficult to measure in a meaningful way; requiring scientific rigour and acumen to gauge progress, guide future development and reassure the public.

Reporting with care and rigour is needed to prevent unnecessary damage to professional or organisational reputations. Such damage affects maligned parties but also can undermine the credibility and acceptability of wider efforts to measure and report on performance. When data are used in the public domain, contributing to the democratic process and social choice, there is little room for spurious associations, erroneous assessments or simplistic measures.

Performance assessment in healthcare is a multi-billion dollar effort – healthcare is one of the most important social services provided to citizens around the world. Trust in published information is an essential feature of high quality healthcare. It is imperative both for accountability and for catalysing continuous improvement that we use assessment frameworks that properly reflect performance, lauding achievements and highlighting areas for renewed efforts to change.

### Supplementary information

**Additional file 1.** Description of selected frameworks.

**Additional file 2.** Framework development stages.

## Figures and Tables

**Fig. 3:**
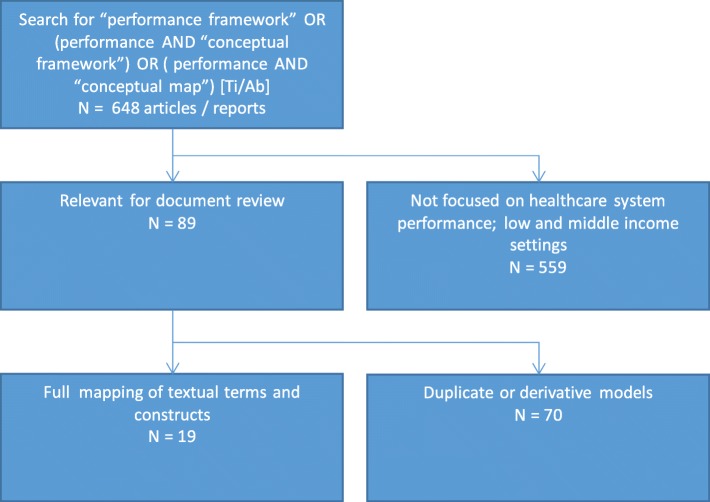
Inclusion and exclusion flowchart

## Data Availability

All data generated or analysed during this study are included in this published article [and its supplementary information files].

## Appendix 2

**Table 3:** Clustering of terms used in the literature according to the framework’s constructs

Constructs	Mapped terms	Constructs	Mapped terms
1. Measurable constructs			
Patients’ needs and expectations	Health care needs / Healthcare needs / Living with illness or disability / chronic care / Risk behaviours / Non-health care determinants of health / Social determinants - genetic endowment, social position, life conditions, physical environment, individual response	Resources and structures	Acquisition of resources / Care cost / Cost / Expenditure / Health workforce, information, medical products vaccines and technologies / Health system resources / Infrastructure / Per capita cost / Resource allocation / Staff
Services	Provision / Volume of care and services / Health promotion / Prevention / Service use	Outcomes	Helping people to recover form episodes of ill health or following injury / Getting better / / Improve health status / Coping with end of life / Enhancing quality of life for people with long-term conditions / Health improvement / Choice /
Processes and functions and context	Organisation and regulation / Climate / Individual engagement / Coordination / Care coordination / Coordinated care / Integrated care / Integration of production / Leadership and governance / Stewardship / Health system design, policy and context / Financing, leadership and governance / Community engagement / Community support / Consensus on values / Context – political, demographic, economic / Context – environmental, demographic, epidemiological, political, legal, economic, social, technological / Work environment		
2. Functional and relational constructs of performance – Patient perspective			
Coverage	Comprehensiveness / Coverage / Financial risk protection / Social and financial risk protection	Accessibility	Access / Access to comprehensive integrated health services / Access to care / Accessibility / Affordability / Cost related problems / Timely / Timeliness / Quality (access safety effect)^a^
Appropriateness	Appropriate treatment / Appropriateness / Conformity to standards / Art of care and respect / Consumer satisfaction / Ensuring that people have a positive experience of care / Experience of care / Individual patient experiences / Patient experience / Patient experience with health services / Patient centredness / Person centred / Responsiveness / Engaged people / Continuity	Safety	Safe care / Safety / Treating and caring for people in a safe environment and protecting them from avoidable harm / Quality, safety and appropriateness of health services / Quality (access safety effectiveness)^a^
3. Functional and relational constructs of performance – static system perspective		4. Functional and relational constructs of performance – dynamic system perspective	
Productivity	Inputs per output unit / Productivity	Adaptability	Adaptation to population health needs / Adjustment to population health needs / Health system innovation and learning capacity / Improve health systems responsiveness / Innovation and learning / System and workforce innovation.
Effectiveness	Clinical effectiveness / Effectiveness / Effective treatment / Effective care / Program effectiveness / Quality (access safety effectiveness)^a^	Resilience	Responsive governance
Efficiency	Cost effectiveness / Efficiency / Efficient allocation of resources / Improve value for money	Sustainability	Attraction of clientele / Creating resources / Employees’ health / Sustainability / Work satisfaction
5. Goal attainment constructs of performance – population perspective			
Equity	Equity / Equity in financing	Impact	Healthy lives / living / Healthy people / Healthy behaviours / Healthy social circumstances / Health / Health status / Improved health (level and equity) / Population health / Preventing people from dying prematurely / Primary prevention / Stakeholder satisfaction.

^a^Mapped to more than one construct
