# Supplementary material for: Combining patient, clinical and system perspectives in assessing performance in healthcare: an integrated measurement framework
Source: BMC Health Serv Res. 2020 Jan 8;20:23. doi: 10.1186/s12913-019-4807-5 (PMC6950882; doi:10.1186/s12913-019-4807-5)
Supplement: Supplementary file 1 — Additional file 1. Description of selected frameworks. [file 12913_2019_4807_MOESM1_ESM.docx]

**Appendix 2 – Description of selected frameworks**

| Frameworks | Objective of framework / performance measurement | Main constructs |
| --- | --- | --- |
| Logic model perspective / production function | | |
| [CIHI] Canadian Institute for Health Information (CIHI) [12] Canada | A framework that assesses performance of the health system against goals and seeks to support efforts to improve. Within the framework, a logic model approach is used with outputs (the delivery of health services to individuals or to populations) produced by the health system considered as intermediate objectives that correspond to the capacity of the health system to provide access to timely, continuous and effective health services. Health system outcomes are measures of actual and perceived goals of the health system for individuals and for the general population. | Access to comprehensive integrated health services / Health protection, health promotion and disease prevention / Appropriateness / Quality /Safety / Efficiency / Improve value for money / Improve health system responsiveness / Effectiveness / Improve health status / Equity / Health system innovation and learning capacity / Leadership and governance / health system resources / efficient allocation of resources / adjustment to population health needs. |
| [ROGS] Productivity Commission Report of Government Services (RoGS)[17], Australia | The framework is used to structure the Commission’s annual Report on Government Services (RoGS). It considers the process through which service providers transform inputs into outputs and outcomes in order to achieve desired objectives. It has a particular focus on outcomes, reflecting demand by governments for outcome-oriented performance information (defined as equity of outcome indicators; program effectiveness; cost effectiveness). This outcome information is supplemented by information on outputs - grouped under ‘equity’, ‘effectiveness’ and ‘efficiency’ headings. | Equity / Effectiveness / Efficiency / Access / Appropriateness / Quality / Program effectiveness / Cost effectiveness |
| [IHP] International Health Partnership (IHP) and World Health Organization (WHO) [34] | A framework developed by IHP and WHO to guide national efforts to monitor and evaluate health systems. Performance is assessed against the goals and objectives of the system – covering relevant disease areas and activities for strengthening health systems. It is structured using categories of: inputs and processes; outputs; outcomes; and impact. | Prevalence risk behaviours and factors / Intervention access and services readiness / Coverage of intervention / Efficiency / Intervention quality / Safety / Responsiveness / Infrastructure / ICT / health workforce / supply chain / information / financing; governance |
| [HSIAO] Hsaio [36] | A framework based on a causal model that seeks to explains a systems outcomes. It focuses particularly on the role of financing in the performance of the healthcare system. There are three components in the model: means (financing methods, structure, regulation, funds allocation, rationing, and institutional arrangements), intermediate outcomes (access, quality of services, equity in financing, efficiency); final goals (health status, financial risk protection, consumer satisfaction). | Access / Quality / Equity in financing / Efficiency / Health status / Financial risk protection / Consumer satisfaction |
| Functionalist model perspective | | |
| [ATUN] Atun and Menabde [21] | Framework identifies health, financial risk protection and consumer satisfaction as ultimate health system goals, with intermediate goals of equity, efficiency (technical and allocative), effectiveness and choice. It asserts that any framework which analyses health system functions also needs to analyse the system context (demographic, economic, political, legal and regulatory, epidemiological, sociodemographic and technological). . Highlights that performance measurement often over-simplifies cause–effect rather than focusing on “dynamic complexity” characterised by networks of relations, feedback loops and non-linearity. | Effectiveness / Efficiency / Equity / Health / Financial risk protection / Consumer satisfaction / Choice |
| [EGIPSS] Évaluation Globale et Intégrée de la Performance des Systèmes de Santé (EGIPSS)[35], Canada | High performance requires a balance across four functional dimensions: Adaptation (acquiring resources, adapting to clients’ needs, receiving community support and developing innovative services); Goal attainment (preventing, diagnosing and treating problems in an effective, efficient and equitable way); Production (providing services in an integrated and organised way that ensures quality, compassion, continuity, accessibility and universality); Maintaining organisational values (achieving consensus on ideals, ensuring two-way communication and fair decision-making, promoting employees’ physical and psychological well-being, work satisfaction, appropriate workload and environment). The dimensions are described as complementary, rather than in conflict and high performance is seen to be based on balance and communication between the four dimensions. | Effectiveness / Efficiency / Equity  Satisfaction of clients and partners / Quantity of care and services / Productivity / Continuity / Ability to adapt and meet client’s needs / Ability to adapt to requirement and tendencies/ Ability to innovate and transform / Capacity to acquire resources / Quality / Capacity to attract the clientele / Consensus with fundamental values / Collaborative climate |
| Goal achievement model perspective | | |
| [AHRQ] National Healthcare Quality Report[13] - Agency for Health Research and Quality, United States of America | The Agency for Healthcare Research and Quality (AHRQ) reports on US healthcare quality and disparities. It is integrated with the National Quality Strategy (NQS) which identifies three aims,based on the IHI framework: 1) Better Care: Improve overall quality, by making health are more patient-centred, reliable, accessible, and safe; 2) Healthy People/Healthy Communities: Improve the health of the population by supporting proven interventions to address behavioural, social, and environmental determinants of health in addition to delivering higher quality care; 3) Affordable Care: Reduce the cost of quality healthcare for individuals, families, employers, and governments. To advance these aims, the NQS focuses on six priorities: a) Patient Safety: b) Person-Centred Care:; c) Care Coordination; d) Effective Treatment; e) Healthy Living: f) Care Affordability: Making quality care more affordable for individuals, families, employers, and governments by developing and spreading new health care delivery frameworks. | Access to healthcare / Care affordability / Care coordination / Effective treatment / Equity (priority populations) / Healthy living / Patient safety / Person- and family-centred care |
| [NHS] National Health Service Outcomes framework[27], United Kingdom | The NHS *Next Stage Review High Quality Care for All* states that high quality care comprises three domains: effectiveness, patient experience and safety. Based on this, the NHS Outcomes Framework was published and included five constructs, 1). Preventing people from dying prematurely; 2) Enhancing quality of life for people with long-term conditions; 3) Helping people to recover from episodes of ill health or following injury; 4) Ensuring that people have a positive experience of care; 5) and Treating and caring for people in a safe environment; and protecting them from avoidable harm. | Review constructs  Effectiveness / Patient experience / Safety  Outcomes framework constructs:  Preventing people from dying prematurely / Enhancing quality of life for people with long-term conditions / Helping people to recover from episodes of ill health or following injury / Ensuring that people have a positive experience of care / Treating and caring for people in a safe environment; and protecting them from avoidable harm |
| [CMWF] The Commonwealth Fund[24], United States of America | A framework to assess national performance, based on IOM definitions of quality. Uses a scorecard, organised into five constructs: 1) health outcomes; 2) quality; 3) access; 4) equity; 5) and efficiency. *Mirror, Mirror* publications[14] use the framework to compare performance of healthcare systems in 11 developed countries. | Healthy Lives / Quality / Access/ Equity  Efficiency  The framework also discusses innovation and improvement where Quality includes four clusters (effective care, coordinated care, safe care, and patient-centred timely care) and  Access includes universal participation and affordability. |
| [OECD] Organisation for Economic Cooperation and Development [9];updated [15] and also used in the Netherlands[16] and | Assessment of healthcare performance evaluates the direct functioning of the delivery system in the context of its stated goals for the level and distribution of benefits and costs of healthcare. The framework identifies healthcare needs (staying healthy, getting better, living with illness or disability and coping with end of life) and domains of quality (effectiveness, safety and responsiveness / patient centredness), accessibility and cost – expenditure). The framework was revised in 2015 with minor changes to the original structure - ‘staying healthy’ was changed to ‘primary/secondary prevention’ in order to distinguish it more clearly from ‘living with illness and disability—chronic care’. The sub-constructs of ‘individual patient experiences’ and ‘integrated care’ were included under the theme of ‘responsiveness’, to pave the ground for future indicator development. | Healthcare needs / Accessibility  Effectiveness / Safety /  Efficiency / Equity / Responsiveness / Patient centredness / Integrated care /  Cost – expenditure |
| [WHO] World Health Organization in Murray and Frenk[10] | The measurement of performance relates goal attainment to the resources available. The framework references the World Health Report 2000 which defined overall health system goals as “improving health and health equity, in ways that are responsive, financially fair, and make the best, or most efficient, use of available resources”. It also noted as an important intermediate goal: achieving greater access to and coverage for effective health interventions, without compromising efforts to ensure provider quality and safety. Improving health encapsulates increasing the average health status and reducing health inequalities. Responsiveness includes respect for persons (including dignity, confidentiality and autonomy of individuals and families to decide about their own health); and client orientation (including prompt attention, access to social support networks during care, quality of basic amenities and choice of provider). Fairness of financial contribution implies that all citizens are protected from financial risks due to healthcare. Variation in performance reflects health system functions such as stewardship, financing, service provision, and resource generation. | Access / Coverage / Equity / Improved efficiency / Safety / Improved health / Social and financial risk protection / Quality /  Responsiveness / Sustainability / System building blocks |
| [PATH] World Health Organisation (WHO) Europe  [28] | Focused at hospital- rather than system-level performance, the PATH framework assesses the extent to which functioning corresponds to societal, patient, and professional norms. It asserts high hospital performance is based on professional competencies in the application of present knowledge, available technologies and resources; efficiency in the use of resources; minimal risk to the patient; responsiveness to the patient; and optimal contribution to health outcomes. Within the health care environment, high hospital performance incorporates responsiveness to community needs and demands, the integration of services in the overall delivery system, and commitment to health promotion. Performance is assessed in relation to the availability of hospitals’ services to all patients irrespective of physical, cultural, social, demographic, and economic barriers. | Clinical effectiveness / Efficiency / Staff / Responsive governance / Safety / Patient-centredness |
| [BELG] Belgian Health System[29] | No underlying definition of performance. The framework is based on previously published frameworks and consultation with local experts and stakeholders. It comprises, three interconnected tiers: health status; non-medical determinants of health; and health promotion and the health system (with four domains of care: preventive care, curative care, long-term care, and end-of-life care). The performance of the health system is grouped into four main dimensions: quality, accessibility, efficiency, and sustainability. The construct of quality is subdivided into effectiveness, appropriateness, safety, patient-centeredness, continuity. Equity, a fifth performance dimension, is considered across all tiers. | Accessibility / Appropriateness / Safety / Effectiveness / Efficiency / Sustainability / Equity / Continuity / Patient centredness / Quality |
| [SCOT] Scotland 2020 Vision ISD [30] | System goals is articulated as “by 2020 everyone is able to live longer healthier lives at home, or in a homely setting.” Performance is defined in terms of three quality ambitions: 1) Safe - no avoidable injury or harm to people from healthcare, and an appropriate, clean and safe environment; 2) Person-centred - mutually beneficial partnerships between patients, their families and those delivering healthcare services which respect individual needs and values and which demonstrates compassion, continuity, clear communication and shared decision-making; 3) Effective - the most appropriate treatments, interventions, support and services will be provided at the right time to everyone who will benefit, and wasteful or harmful variation will be eradicated. | Safe / Person-centred / Effective |
| [CHEN] Ministry of Health and Welfare, Taiwan[31] | This framework draws on PATH [28] and OECD[9], to build a matrix of five quality dimensions and three application domains (hospital wide, non-surgical, and surgical) | Safety / Clinical effectiveness / Patient centredness / Efficiency / Staff orientation |
| [IHI] Institute for Healthcare Improvement, United States of America[32] | The IHI Triple Aim is a framework that considers health system performance in terms of three key goals: 1) Improving patient experience of care (including quality and satisfaction); 2) Improving the health of populations; 3) Reducing the per capita cost of health care. | Experience of care / Population health / Per capita cost / |
| [LANG] Langton et al, Canada[33 | This framework guides assessment of the extent to which primary care systems meet the needs of patients. Informed by a review of existing measurement systems and accepted conceptual frameworks that articulate features of high-quality primary care systems, the framework incorporates patient or population segments that represent different primary care needs (all, healthy, at risk, one chronic condition, multiple chronic conditions, advanced complex chronic conditions), in a matrix structure with measures that reflect performance domains. | Access / Comprehensiveness / Continuity / Coordination / Effectiveness / Efficiency / Equity / Person-centredness / Safety / Service use / Cost / Patient needs |
| [IOM] Institute of Medicine [26]  United States of America | System goals are articulated as: to continually reduce the burden of illness, injury and disability and to improve the health and functioning of the people of the United States. Six key dimensions of performance are identified: safe (avoiding injuries to patients from the care that is intended to help them); effective (providing services based on scientific knowledge to all who could benefit and refraining from providing services to those not likely to benefit - avoiding underuse and overuse, respectively); patient-centred (providing care that is respectful of and responsive to individual patient preferences, needs, and values and ensuring that patient values guide all clinical decisions); timely (reducing waits and sometimes harmful delays for both those who receive and those who give care); efficient (avoiding waste, including waste of equipment, supplies, ideas, and energy); .equitable (providing care that does not vary in quality because of personal characteristics such as gender, ethnicity, geographic location, and socioeconomic status) | Safe / Effective / Patient-centred / Timely / Efficient / Equitable |
| [VITAL] Institute of Medicine[5] United States of America | A set of measures that draws of the IOM conceptualisation of quality. The framework seeks to provide a reliable reflection of health and health care at national, state, local, and institutional level. The measurement and reporting framework aims to draw sustained attention to what is important, focus on results rather than processes, reduce the number of measurements required for reporting purposes, increase flexibility and capacity for innovation at the local and institutional levels, and enhance the effectiveness and efficiency of system performance.. | Healthy people / Care quality / Care cost / Engaged people / Length of life / Quality of life / Healthy behaviours / Healthy social circumstances / Prevention / Access to care / Safe care / Appropriate treatment / Person-centred care / Affordability / Sustainability . Individual engagement / Community engagement |
