# Supplementary material for: Combining patient, clinical and system perspectives in assessing performance in healthcare: an integrated measurement framework
Source: BMC Health Serv Res. 2020 Jan 8;20:23. doi: 10.1186/s12913-019-4807-5 (PMC6950882; doi:10.1186/s12913-019-4807-5)
Supplement: Supplementary file 2 — Additional file 2. Framework development stages. [file 12913_2019_4807_MOESM2_ESM.docx]

**Appendix 4 – Framework development stages**

**Iteration 2:** A reconfiguration sought to move away from linearity. Changing the orientation, revealed the missing constructs of appropriateness. Impact and productivity are noted but less prominently than other constructs. Coverage was not included. Reproduced from the NSW Bureau of Health Information (2015) [59].

**Iteration 1:** This model established the key principle that while Donabedian constructs of structures, processes and outcomes are measurable, they do not necessarily reflect performance. Performance is conceptualised as a relative construct - assessed with derived measures.

**
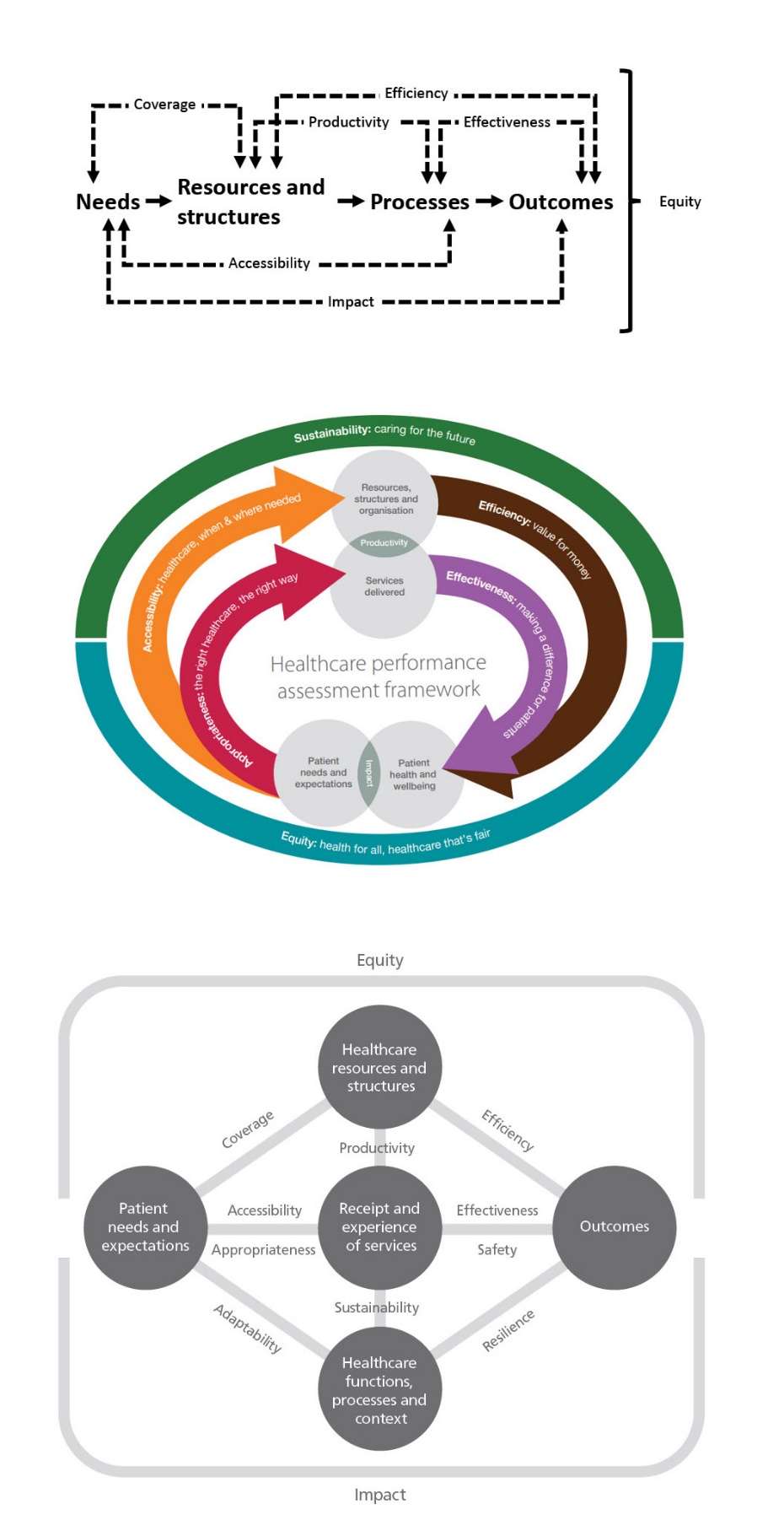
**

**Iteration 3:** Further reconfiguration and the addition of a node focused on healthcare functions, processes and context revealed additional performance constructs of coverage, adaptability, resilience, safety. Impact and productivity become more prominent. This iteration is conceptually grounded and mapping performance constructs used in the literature showed the model to be comprehensive in scope. In practical measurement terms, not all constructs are easily populated.
